# Motivation and job satisfaction among medical and nursing staff in a Cyprus public general hospital

**DOI:** 10.1186/1478-4491-8-26

**Published:** 2010-11-16

**Authors:** Persefoni Lambrou, Nick Kontodimopoulos, Dimitris Niakas

**Affiliations:** 1 Faculty of Social Sciences, Hellenic Open University, Bouboulinas 57, 26222, Patras, Greece; 2 Nicosia General Hospital, Nicosia, Cyprus

## Abstract

**Background:**

The objective of this study was to investigate how medical and nursing staff of the Nicosia General Hospital is affected by specific motivation factors, and the association between *job satisfaction *and *motivation*. Furthermore, to determine the motivational drive of socio-demographic and job related factors in terms of improving work performance.

**Methods:**

A previously developed and validated instrument addressing four work-related motivators (*job attributes, remuneration, co-workers and achievements*) was used. Two categories of health care professionals, medical doctors and dentists (N = 67) and nurses (N = 219) participated and motivation and job satisfaction was compared across socio-demographic and occupational variables.

**Results:**

The survey revealed that *achievements *was ranked first among the four main motivators, followed by *remuneration*, *co-workers *and *job attributes*. The factor *remuneration *revealed statistically significant differences according to gender, and hospital sector, with female doctors and nurses and accident and emergency (A+E) outpatient doctors reporting greater mean scores (p < 0.005). The medical staff showed statistically significantly lower job satisfaction compared to the nursing staff. Surgical sector nurses and those >55 years of age reported higher job satisfaction when compared to the other groups.

**Conclusions:**

The results are in agreement with the literature which focuses attention to management approaches employing both monetary and non-monetary incentives to motivate health care professionals. Health care professionals tend to be motivated more by intrinsic factors, implying that this should be a target for effective employee motivation. Strategies based on the survey's results to enhance employee motivation are suggested.

## Background

Motivation can be defined as the processes that account for an individual's intensity, direction and persistence of effort toward attaining a goal [1]. In most cases motivation stems from a need which must be fulfilled, and this in turn leads to a specific behavior. Fulfillment of needs results in some type of reward, which can be either intrinsic or extrinsic. The former are derived from within the individual, e.g. taking pride and feeling good about a job well-done, whereas the latter pertain to rewards given by another person [2]. Job satisfaction, on the other hand, is defined as a pleasurable or positive emotional state, resulting from the appraisal of one's job or job experiences.

Psychologists have studied human motivation extensively and have formulated a variety of theories about what motivates people. Needs-based theories include Maslow's hierarchy of need, Aldersfer's theory, Herzberg's two factor theory and McClelland's acquired needs theory. Another approach focuses on external factors and their role in understanding employee motivation (e.g. Skinner's reinforcement theory). Theories based on intrinsic factors focus on internal thought processes and perceptions about motivation (e.g. Adam's equity theory, Vroom's expectancy theory, Locke's goal setting theory) [2].

In the health care field, attaining health objectives in a population depends to a large extent on the provision of effective, efficient, accessible, viable and high-quality services. The health workforce, present in sufficient numbers and appropriately allocated across different occupations and geographical regions is arguably the most important input in a unique production process and has a strong impact on overall health system performance [3]. The lack of explicit policies for human resource management has produced, in most countries, imbalances that threaten the capacity of health care systems to attain their objectives [4].

The workforce in the health sector has specific features that cannot be ignored and motivation can play an integral role in many of the compelling challenges facing healthcare today [5]. In this area, the task of motivation is exacerbated by i) the nature of the economic relationship between those using the system and the system itself (physicians, patients and hospitals) and ii) the heterogeneity of the workforce to be managed [6]. Health organizations are faced with external pressures that cannot be effectively met without appropriate adjustments to the workforce and the development of the workforce thus appears to be a crucial part of the health policy development process [7].

Cyprus has been a member of EU since 2004. The health system is a typical South Mediterranean system, with provision of health care services from both the private and public sectors. Cyprus has the second lowest share of public expenditure to total health expenditure (43.2%), with the latter approximating 6% of the GDP. Health indicators are above the EU average, with life expectancy at 78.8 and 82.4 years for males and females respectively [8]. The public health sector system consists of eight hospitals and a number of primary health care centers around the country. The hospitals offer services for primary, secondary and tertiary care. The health system is currently undergoing major changes, and in 2011 a new General Health System is expected to be implemented, which is to be based on contributions by the employers and the employees.

Human resources management practices in the public health sector in Cyprus are centralized. Recruitment and selection is conducted by the Civil Service Committee (appointed by the President of the Republic every five years). Consequently, hospital management is practically unable to ensure employee motivation, due to a lack of autonomy. There is a problem of split accountability of personnel, as doctors and other health professionals are accountable centrally to the Medical Services, dentists to the Dental Services, psychiatrists to the Mental Health Services and administrative officers to the Ministry of Finance and other Ministries. All public sector physicians are salaried employees of the Ministry of Health and belong to a centralized civil service staffing system that allocates them to posts based on defined needs. A plan to make hospitals autonomous is now in place in the Ministry of Health, and it is emphasized that the development of this autonomy will be the prerequisite of the implementation of the new General Health System [9].

Although employee motivation is a significant element of health systems' performance, it is largely understudied [10]. The purpose of this study was to investigate: i) how medical and nursing staff of the Nicosia General Hospital is affected by specific motivation factors, ii) the association between *job satisfaction *and *motivation *iii) the motivational drive of socio-demographic and job related factors in terms of improving performance in this hospital. A validated instrument [11] addressing four work-related motivators (*job attributes, remuneration, co-workers and achievements*) was used. Two categories of health care professionals, doctors (including dentists) and nurses working in the hospital participated. *Job satisfaction *was cross-related to these motivational factors. The main focus was on potential differences between the two categories of professionals.

## Methods

### Instrument

An instrument developed for measuring motivation based on Maslow's and Herzberg's theories was used in the present study. It consists of 19 items which are grouped under four distinct motivational factors. The *job attributes *factor encompasses 7 items: authority, goals, creativity opportunities, clear duties, job control, skill exploitation and decision-making. The *remuneration *factor encompasses 4 items: salary, environment, retirement/pension and absenteeism. The *co-workers *factor encompasses 5 items: teamwork, job pride, appreciation, supervisor and fairness. The *achievements *factor encompasses 3 items: job meaningfulness, earned respect and interpersonal relationships [11]. All items are neutrally phrased as *"In your case, how important is..*.... *for increasing your will to perform better at work?"*. Responses are provided on a five-point unipolar adjective scale, in which 1 corresponds to *"not at all"*, 2 to *"a little bit"*, 3 to *"moderately"*, 4 to *"very" *and 5 to *"extremely*. The survey included a single question relevant to job satisfaction which was measured on a 1-5 scale as well. The most frequently argued advantages of single item measures for measuring overall *job satisfaction *are brevity, increased face validity, high correlation with multi-item satisfaction measures and increased sensitivity in measuring changes in *job satisfaction *[12,13]. Socio-demographic data on age, gender, education, and work-related data such as years in service, department and managerial position were also collected.

### Sample and data collection

The present study was conducted in the Nicosia General Hospital which is the largest on the island with a capacity of 414 beds. Its operation started in 2007 and today it employs 161 doctors and 770 nurses. The questionnaire was randomly distributed to 50% of the medical doctors (including dentists) and nurses between November and December 2008. In total 67 doctors and 219 nurses responded to the questionnaire, with an overall response rate of 76.6%. Clinical departments were grouped into 4 sectors: accident and emergency (A+E) outpatients' clinics constituted one sector, general surgery, angiothoracic, anesthetic, dental, E.N.T., plastic surgery, neurosurgery, orthopedic, urology and the nursing personnel of the operating theatres constituted the surgical sector, I.T.U., medical, cardiology, renal, oncology, dermatology and the chest clinic constituted the medical sector and X-ray, pathology and forensic medicine constituted the laboratory sector.

### Analysis

The sample was analyzed as a whole and by professional subgroup. For each motivation factor, summated scores were calculated on a 1-5 scale, with higher scores corresponding to higher motivation to perform better by that particular factor. Parametric t-test and ANOVA were used for comparisons according to gender, education, age and job-related variables such as years in service, department and if the respondent appraised subordinates. Multivariate analyses, with each motivation factor the dependent variable, and sociodemographic, work-related and job satisfaction variables as independent predictors were conducted. Internal consistency reliability was tested via Cronbach's alpha coefficient and compared with the respective values observed during the development of the instrument [11]. All analyses were performed with SPSS version 15.0 (SPSS Inc., Chicago IL).

## Results

The sample frequency distribution, according to demographic and work-related variables, is shown in Table 1. The majority of the respondents were female (68.5%) mostly due to the large number of female nurses, and the mean time serving in the public health sector was 14.1 years. By subgroup, doctors were predominantly males (64.2%), whereas nurses were females (78.5%). The age distribution was: 12.6% under 25 years old, 25.9% between 25 and 35, 22.0% between 36 and 45, 29.7% between 46 and 55 and 9.8% over 55. Regarding hospital sector, 39.9% were from the medical sector, 31.5% from the surgical sector, 26.2% from A+E outpatients sector and 2.4% were from the Lab sector. Only 23.4% of the respondents were responsible for managing other people.

**Table 1:** Overall and sub-sample frequency distribution by demographic and job-related variables

Demographic variables	Overall (N = 286)	Doctors/ Dentists (N = 67)	Nurses (N = 219)
Gender			
Male	90 (31.5%)	43 (64.2%)	47 (21.5%)
Female	196 (68.5%)	24 (35.8%)	172 (78.5%)

Sector			
Medical	114 (39.9%)	19 (28.4%)	95 (43.4%)
Surgical	90 (31.5%)	20 (29.8%)	70 (31.9%)
A+E/Outpatients	75 (26.2%)	21 (31.4%)	54 (24.7%)
Laboratory	7 (2.4%)	7 (10.4%)	-

Age group			
<25	36 (12.6%)	-	36 (16.4%)
25-35	74 (25.9%)	4 (5.9%)	70 (31.9%)
36-45	63 (22.0%)	21 (31.4%)	42 (19.3%)
46-55	85 (29.7%)	25 (37.3%)	60 (27.4%)
>55	28 (9.8%)	17 (25.4%)	11 (5.0%)

Years in public service			
<5	83 (29.0%)	16 (23.9%)	67 (30.6%)
5-10	50 (17.5%)	17 (25.4%)	33 (15.1%)
11-20	70 (24.5%)	20 (29.9%)	50 (22.9%)
21-30	58 (20.3%)	13 (19.3%)	45 (20.5%)
>30	25 (8.7%)	1 (1.5%)	24 (10.9%)

Management position			
Yes	67 (23.4%)	18 (26.8%)	49 (22.4%)
No	219 (76.6%)	49 (73.2%)	170 (77.6%)

The mean scores for each motivating factor are shown in Table 2. The highest ranked motivator was *achievements*, which was significantly higher than all the others both for the overall sample, and by professional subgroup. The second highest ranked motivator overall was *remuneration*, however doctors ranked *co-workers *as the second strongest motivating factor. The scores for *remuneration *were statistically significantly different overall and by subgroup. The lowest ranked motivator by both groups was *job attributes*. The four scales showed high internal consistency reliability and Cronbach's alpha coefficient was found to be in accordance with the respective developmental values [11]. Specifically, alpha was 0.838 (compared to 0.822 reported in the validation study) for *remuneration*, 0.744 (0.782) for *achievements*, 0.847 (0.826) for *co-workers *and 0.897 (0.901) for *attributes*. On the other hand, the single-item *satisfaction *scale showed a moderate correlation with each of the motivation factors, with Pearson's r ranging between 0.303-0.382 (*P *< 0.001).

**Table 2:** Mean scores^1 ^(SD) by motivating factor and job satisfaction for the entire sample and by professional subgroup

	Overall (N = 286)	Doctors/ Dentists (N = 67)	Nurses (N = 219)
Remuneration	3.65 (1.02)	3.58 (1.07)	3.67 (1.01)
Job attributes	3.37 (0.98)	3.55 (1.03)	3.32 (0.96)
Coworkers	3.59 (0.99)	3.67 (1.07)	3.57 (0.96)
Achievements	4.18 (0.85)	4.26 (0.85)	4.16 (0.85)
Job satisfaction	3.21 (1.01)	3.07 (1.10)	3.25 (0.97)

^1 ^Reported on a 1-5 scale with higher values corresponding to higher motivation

Scores by demographic and work-related variables for the *remuneration *factor, which encompasses extrinsic motivators such as salary, benefits, pension and vacation schemes are shown in Table 3. Interestingly, the motivating effect of *remuneration *was significantly different by professional category, and appeared to be influenced by gender and by sector (for doctors). Specifically, female doctors reported being motivated significantly (*P *< 0.05) more by *remuneration *than their male counterparts. Furthermore, doctors working in the A+E outpatients sector were motivated more by *remuneration *(*P *< 0.05) than those working elsewhere.

**Table 3:** Mean scores^1 ^(SD) by demographic and job-related variables for the REMUNERATION motivator

Demographic variables	Overall (N = 286)	Doctors/ Dentists (N = 67)	Nurses (N = 219)
Gender			
Male	3.36 (0.99)	3.35 (1.09)	3.36 (0.90)
Female	3.78 (1.01)	4.01 (0.90)	3.75 (1.02)
*P-sig.^2^*	***0.010***	***0.015***	***0.020***

Sector			
Medical	3.53 (1.15)	2.93 (1.01)	3.65 (1.14)
Surgical	3.78 (0.94)	3.76 (1.05)	3.78 (0.92)
A+E/Outpatients	3.65 (0.89)	3.92 (0.98)	3.54 (0.84)
Laboratory	3.85 (0.89)	3.85 (0.89)	-
*P-sig.^3^*	*0.365*	***0.014***	*0.420*

Age group			
<25	3.93 (1.09)	-	3.93 (1.09)
25-35	3.59 (1.01)	3.50 (1.67)	3.60 (0.97)
36-45	3.61 (0.96)	3.38 (1.11)	3.72 (0.67)
46-55	3.68 (1.02)	4.04 (0.79)	3.54 (1.07)
>55	3.41 (1.07)	3.20 (1.06)	3.72 (1.06)
*P-sig.^3^*	*0.328*	*0.055*	*0.433*

Years in public service			
<5	3.68 (1.11)	3.48 (1.45)	3.73 (1.05)
5-10	3.41 (1.15)	3.41 (1.15)	3.81 (0.96)
11-20	3.64 (0.83)	3.72 (0.69)	3.61 (0.88)
21-30	3.55 (1.02)	3.63 (1.09)	3.52 (1.01)
>30	3.73 (1.22)	5.00 (-)	3.67 (1.22)
*P-sig.^3^*	*0.936*	*0.621*	*0.747*

Management position			
Yes	3.75 (0.99)	3.52 (1.04)	3.84 (0.97)
No	3.61 (1.03)	3.61 (1.08)	3.62 (1.01)
*P-sig.^2^*	*0.333*	*0.777*	*0.178*

^1 ^Reported on a 1-5 scale with higher values corresponding to higher motivation

^2 ^According to t-test

^3 ^According to ANOVA

*Job attributes *encompasses intrinsic motivators such as decision-making, creativity and skill exploitation. This factor appears (Table 4) to motivate doctors more than nurses and specifically the respondents in managerial positions. Female doctors reported being more motivated by this factor than their male colleagues. Those working in the A+E outpatients sector, nurses >55 years old and those working >30 years scored higher. Interestingly, only one statistically significant difference was observed in the analyses for this motivator, and specifically nurses in management positions compared to those not having such responsibilities (*P *= 0.049).

**Table 4:** Mean scores^1 ^(SD) by demographic and job-related variables for the JOB ATTRIBUTES motivator

Demographic variables	Overall (N = 286)	Doctors/ Dentists (N = 67)	Nurses (N = 219)
Gender			
Male	3.23 (1.02)	3.38 (1.11)	3.10 (0.92)
Female	3.44 (0.96)	3.86 (0.81)	3.38 (0.96)
*P-sig.^2^*	*0.108*	*0.065*	*0.086*

Sector			
Medical	3.32 (1.00)	3.29 (0.94)	3.32 (1.01)
Surgical	3.46 (0.92)	3.67 (1.13)	3.40 (0.85)
A+E/Outpatients	3.34 (1.05)	3.68 (1.15)	3.20 (0.99)
Laboratory	3.55 (0.60)	3.55 (0.60)	-
*P-sig.^3^*	*0.717*	*0.625*	*0.535*

Age group			
<25	3.32 (0.94)	-	3.32 (0.94)
25-35	3.23 (1.00)	3.10 (1.40)	3.24 (0.99)
36-45	3.39 (1.06)	3.39 (1.31)	3.39 (0.93)
46-55	3.42 (0.50)	3.88 (0.65)	3.23 (0.93)
>55	3.62 (1.01)	3.38 (0.99)	4.00 (0.95)
*P-sig.^3^*	*0.458*	*0.249*	*0.157*

Years in public service			
<5	3.28 (0.98)	3.30 (1.17)	3.27 (0.94)
5-10	3.43 (1.04)	3.48 (1.27)	3.40 (0.93)
11-20	3.44 (0.90)	3.80 (0.58)	3.30 (0.97)
21-30	3.33 (0.93)	3.46 (1.05)	3.30 (0.90)
>30	3.46 (1.19)	5.00 (-)	3.40 (1.17)
*P-sig.^3^*	*0.089*	*0.383*	*0.963*

Management position			
Yes	3.62 (0.89)	3.78 (0.99)	3.55 (0.86)
No	3.30 (0.99)	3.47 (1.05)	3.25 (0.98)
*P-sig.^2^*	*0.089*	*0.277*	***0.049***

^1 ^Reported on a 1-5 scale with higher values corresponding to higher motivation

^2 ^According to t-test

^3 ^According to ANOVA

The *co-workers *motivator (Table 5) refers to professional relationships with supervisors and colleagues as a source of satisfaction and motivation. The scores for this factor were practically identical in all groups and no statistically significant differences were observed. This motivator was ranked second among doctors and third among nurses. Respondents working in the surgical and A+ E outpatient sectors reported being more motivated by these aspects, than those working in the other sectors. Well-established professional relationships motivated nurses in managerial positions and those aged >55 years old.

**Table 5:** Mean scores^1 ^(SD) by demographic and job-related variables for the COWORKERS motivator

Demographic variables	Overall (N = 286)	Doctors/ Dentists (N = 67)	Nurses (N = 219)
Gender			
Male	3.52 (1.02)	3.65 (1.12)	3.40 (0.93)
Female	3.62 (0.97)	3.70 (1.00)	3.61 (0.97)
*P-sig.^2^*	*0.401*	*0.836*	*0.181*

Sector			
Medical	3.55 (1.00)	3.47 (0.95)	3.56 (1.02)
Surgical	3.63 (0.89)	3.78 (1.20)	3.59 (0.79)
A+E/Outpatients	3.61 (1.08)	3.78 (1.08)	3.54 (1.08)
Laboratory	3.57 (1.09)	3.57 (1.09)	-
*P-sig.^3^*	*0.943*	*0.779*	*0.965*

Age group			
<25	3.56 (0.85)	-	3.56 (0.85)
25-35	3.59 (0.93)	3.55 (1.32)	3.59 (0.92)
36-45	3.54 (1.06)	3.42 (1.21)	3.60 (0.99)
46-55	3.55 (1.03)	3.92 (0.95)	3.40 (1.03)
>55	3.87 (1.02)	3.63 (1.02)	4.23 (0.94)
*P-sig.^3^*	*0.649*	*0.488*	*0.125*

Years in public service			
<5	3.54 (0.92)	3.60 (1.23)	3.53 (0.84)
5-10	3.77 (0.98)	3.65 (1.22)	3.83 (0.85)
11-20	3.55 (1.03)	3.65 (0.99)	3.51 (1.08)
21-30	3.48 (0.92)	3.70 (1.01)	3.41 (0.90)
>30	3.77 (1.24)	5.00 (-)	3.72 (1.24)
*P-sig.^3^*	*0.469*	*0.814*	*0.344*

Management position			
Yes	3.66 (1.05)	3.62 (1.14)	3.68 (1.03)
No	3.57 (0.97)	3.68 (1.06)	3.54 (0.94)
*P-sig.^2^*	*0.507*	*0.822*	*0.367*

^1 ^Reported on a 1-5 scale with higher values corresponding to higher motivation

^2 ^According to t-test

^3 ^According to ANOVA

Scores for the *achievements *factor, which refers to intrinsic motivators such as pride, appreciation, respect and social acceptance are shown in Table 6. This motivator was ranked as the strongest by both doctors and nurses. However, the doctor and nurse subgroup analyses did not reveal any statistically significant differences. Regarding the scores for *job satisfaction *(Table 7), the medical staff presented statistically significant lower ratings compared to the nursing staff. The subgroup analyses showed statistically significant differences in 4 cases. The most determining variable was age, which was associated with higher *satisfaction *for doctors in the 46-55 age group and for nurses >55 years old (*P *< 0.01). More years in public service was reported as a significant source of *satisfaction *for nurses (*P *= 0.01), as was working in the surgical sector as well (*P *< 0.05).

**Table 6:** Mean scores^1 ^(SD) by demographic and job-related variables for the ACHIEVEMENTS motivator

Demographic variables	Overall (N = 286)	Doctors/ Dentists (N = 67)	Nurses (N = 219)
Gender			
Male	4.13 (0.83)	4.19 (0.94)	4.08 (0.73)
Female	4.20 (0.85)	4.38 (0.67)	4.18 (0.87)
*P-sig.^2^*	*0.517*	*0.376*	*0.489*

Sector			
Medical	4.84 (0.81)	4.26 (0.52)	4.11 (0.85)
Surgical	4.32 (0.85)	4.38 (1.03)	4.30 (0.80)
A+E/Outpatients	4.08 (0.90)	4.15 (0.95)	4.04 (0.88)
Laboratory	4.23 (0.87)	4.23 (0.87)	-
*P-sig.^3^*	*0.288*	*0.876*	*0.207*

Age group			
<25	4.31 (0.85)	-	4.31 (0.85)
25-35	4.13 (0.86)	4.00 (0.98)	4.14 (0.96)
36-45	4.15 (0.81)	4.26 (0.87)	4.09 (0.79)
46-55	4.11 (0.87)	4.29 (0.80)	4.04 (0.90)
>55	4.42 (0.79)	4.27 (0.95)	4.66 (0.33)
*P-sig.^3^*	*0.408*	*0.941*	*0.166*

Years in public service			
<5	4.22 (0.81)	4.16 (0.76)	4.23 (0.83)
5-10	4.37 (0.75)	4.43 (0.88)	4.34 (0.68)
11-20	4.02 (0.89)	4.20 (0.81)	3.96 (0.92)
21-30	4.08 (0.86)	4.20 (1.05)	4.05 (0.81)
>30	4.36 (0.96)	5.00 (-)	4.33 (0.97)
*P-sig.^3^*	*0.147*	*0.790*	*0.162*

Management position			
Yes	4.24 (0.90)	4.12 (1.10)	4.29 (0.83)
No	4.16 (0.83)	4.31 (0.75)	4.12 (0.85)
*P-sig.^2^*	*0.487*	*0.442*	*0.221*

^1 ^Reported on a 1-5 scale with higher values corresponding to higher motivation

^2 ^According to t-test

^3 ^According to ANOVA

**Table 7:** Mean scores^1 ^(SD) by demographic and job-related variables for JOB SATISFACTION

Demographic variables	Overall (N = 286)	Doctors/ Dentists (N = 67)	Nurses (N = 219)
Gender			
Male	3.18 (1.07)	3.14 (1.12)	3.23 (1.05)
Female	3.21 (0.98)	2.96 (1.08)	3.26 (0.97)
*P-sig.^2^*	*0.813*	*0.524*	*0.893*

Sector			
Medical	3.14 (1.02)	3.26 (1.14)	3.11 (1.00)
Surgical	3.34 (0.98)	2.70 (1.08)	3.52 (0.88)
A+E/Outpatients	3.12 (1.02)	3.09 (1.13)	3.12 (0.99)
Laboratory	3.57 (0.78)	3.57 (0.78)	*-*
*P-sig.^3^*	*0.307*	*0.236*	***0.015***

Age group			
<25	3.19 (0.95)	-	3.19 (0.95)
25-35	2.91 (1.04)	2.25 (0.95)	2.95 (1.04)
36-45	3.03 (0.96)	2.66 (1.11)	3.21 (0.84)
46-55	3.52 (0.95)	3.52 (1.04)	3.53 (0.92
>55	3.42 (0.99)	3.11 (0.99)	3.90 (0.83)
*P-sig.^3^*	***0.001***	***0.023***	***0.002***

Years in public service			
<5	3.06 (1.06)	3.12 (1.20)	3.04 (1.03)
5-10	2.96 (1.00)	2.88 (1.16)	3.00 (0.93)
11-20	3.24 (0.95)	3.35 (0.98)	3.20 (0.94)
21-30	3.31 (0.90)	2.84 (1.14)	3.44 (0.78)
>30	3.88 (0.97)	3.00 (-)	3.91 (0.97)
*P-sig.^3^*	***0.002***	*0.684*	***0.010***

Management position			
Yes	3.40 (1.02)	3.16 (1.09)	3.48 (0.86)
No	3.15 (1.02)	3.04 (1.11)	3.18 (1.00)
*P-sig.^2^*	*0.074*	*0.683*	*0.053*

^1 ^Reported on a 1-5 scale with higher values corresponding to higher motivation

^2 ^According to t-test

^3 ^According to ANOVA

In a series of multivariate analyses, each motivational factor was regressed against socio-demographic variables (gender, age), work related variables (years in service, managing people) and *job satisfaction*, and the results are presented in Table 8. Reporting high satisfaction from work was positively and significantly associated with higher scores in all motivational factors, for both professional categories. The only motivator significantly affected by variables other than satisfaction was *remuneration*. Specifically in the nurses group, stronger motivation by *remuneration *aspects was associated with female gender, fewer years in service and occupying a managerial position, whereas in the doctors subgroup with female gender. This variable (i.e. gender) was also a significant predictor for motivation by *job attributes *for doctors.

**Table 8:** Multivariate analyses for motivation factors by professional category

	B Coefficient *(p value)*							
	
	JOB ATTRIBUTES		REMUNERATION		CO-WORKERS		ACHIEVEMENTS	
			
Model	Doctors/Dentists	Nurses	Doctors/Dentists	Nurses	Doctors/Dentists	Nurses	Doctors/Dentists	Nurses
Constant	1.782 (*0.001)*	2.180 *(<0.001)*	1.699 (*0.002)*	1.676 *(<0.001)*	2.732 *(<0.001)*	2.242 *(<0.001)*	3.589 *(<0.001)*	3.128 *(<0.001)*
Female	0.548 (*0.028)*		0.710 (*0.006)*	0.369 (*0.018)*				
Age (10-year groups)								
Years in service				-0.022 (*0.001)*				
Management position				0.389 (*0.030)*				
Job satisfaction	0.335 (*0.003)*	0.351 *(<0.001)*	0.301 (*0.008)*	0.364 *(<0.001)*	0.306 (*0.010)*	0.409 *(<0.001)*	0.219 (*0.021)*	0.318 *(<0.001)*

R^2^=	0.152	0.124	0.158	0.146	0.058	0.168	0.066	0.130

## Discussion

Cyprus's health system faces challenges such as accession into the E.U., introduction of a new general health insurance system and the introduction of advanced medical technology. Changes in health care are continuous and at an accelerated pace; with these changes the need for more inspiring employees is emerging. How does one motivate employees in the face of increased demands, particularly when they are being asked to meet these demands with fewer resources? Motivation plays an integral role in many of the compelling challenges facing the health workforce today. Motivation theories classify sources of motivation into those intrinsic and those extrinsic to work. Thus, exploration of the motivating factors for today's health workforce may yield valuable insight into many of the challenges facing modern hospitals. Meeting the needs and achieving the goals of both the employee and the organization is the cornerstone of job satisfaction and this is of crucial importance for management, as it is correlated with the upgrading of the quality of the services provided [14].

One objective of this study was to investigate how medical and nursing staff of the largest public hospital in Cyprus, namely the Nicosia General Hospital, was affected by four specific motivation factors. A validated questionnaire based on Maslow's needs theory and Herzberg's two factor theory was used. The motivational factors investigated were *job attributes*, *remuneration*, *co-workers *and *achievements*. In total, 286 employees responded to the questionnaire, 67 medical doctors (including 8 dentists) and 219 nurses. The survey revealed that *achievements *were ranked as first among the four main motivators, followed by *remuneration*, *co-workers *and *job attributes*. *Achievements*, which is an intrinsic factor, was the main motivator in both the doctor and nurse subgroups. Thus, delegation of authority, recognition of personnel efforts, opportunities for promotion and the job enrichment must be a part of the hospital human resource strategy [15-17]. Aspects encompassed in *remuneration *also appeared to be very important to the respondents. Nevertheless, due to the strict legal remuneration framework in the public service, any deviations regarding this issue are limited.

A recent Greek study having used the same instrument resulted in similar findings [18], i.e. *achievements *once again was the most profound motivator in the three professional subgroups investigated (doctors, nurses and office workers) and in the overall sample (N = 1353). Agreement was also observed in that *remuneration *and *co-workers *followed closely, and *job attributes *again had the least influence on motivation. It is worth mentioning however that the Greek study was focused on comparing motivation in the public and private health care sectors, implying that its results may not be directly comparable to the present study.

Our results are in line with findings from similar studies in which different data collection methods were employed. One study conducted in two very different cultural and socio-economic environments (Jordan and Georgia) reported self-efficacy, pride and values as important motivational parameters [19], i.e. constructs which fall under our *achievements *factor. Another study conducted in two African countries, namely Benin and Kenya, which used qualitative interviews also demonstrated the importance of non-financial incentives in increasing the motivation of health professionals [20]. Qualitative studies from Vietnam [21] and Tanzania [22] showed motivation to be influenced by both financial and non-financial incentives and motivating factors were appreciation by managers, colleagues and the community, a stable job and income and training. A study from Mali based on a mixed-methods approach showed the importance of adapting or improving upon performance management strategies to influence staff motivation [23]. Finally, a German study addressing satisfaction among physicians clearly showed that work and profession related variables were more important than financial situation [24].

Several interesting points worth mentioning arose from this study. For example, female doctors and nurses reported being more motivated by remuneration compared to their male counterparts. This same factor was also significant for accident/emergency outpatient doctors, but not nurses. A possible explanation may be that this is the only category of doctors working under a rotational shift system (compared to all of the nurses), and that extra wages may be a way of overcoming their potential dissatisfaction. Another interesting observation was that job satisfaction was higher, (implying higher motivation as well) in nurses in managerial positions and those aged >55 years. Nurses overall showed higher satisfaction from their work compared to doctors, a finding which is interesting yet contradictory to results from a recent Greek study reporting that nurses were less satisfied than other health care professionals [25], which may be explained by the fact the latter study was conducted in a mental health setting.

The results from this study could be potentially important in terms of human resource management policies to be applied in this particular setting. As previously mentioned, intrinsic motivators (e.g. work meaningfulness, strong interpersonal relationships, respect etc.) have been shown to have a positive effect on service quality, implying that the hospital's administration could start its effort to motivate doctors and nurses. Frequent goal-setting meetings with their representatives might be a start, i.e. a type of *quality circle *to mutually identify, analyze and solve work-related problems in order to improve the performance of the hospital, and motivate and enrich the work of employees. In this line of discussion, the Ministry of health's plan to make hospitals autonomous could be supported by a management-by-objectives strategy, aiming first and foremost to exploit the existing workforce by attempting to satisfy and motivate it.

In an attempt to cross-relate *motivation *with *job satisfaction*, the latter was assessed via a single question with a five-point response scale. The medical staff presented statistically significant lower ratings in *job satisfaction *compared to the nursing staff, a finding not in accordance with findings from another recent study in Greece conducted in the mental health care sector [25]. Our findings require further exploration, perhaps via a larger sample of health care professionals. *Job satisfaction *was statistically significantly higher for surgical sector nurses and those in the >55 age group. Similar results have been observed in recent *job satisfaction *studies [26-28].

The *satisfaction scale *showed a moderate correlation with all motivational factors, with Pearson's r ranging between 0.303-0.382. Although the association was significant (*P *< 0.001), these correlations mean that *job satisfaction *accounts for only 8.9%-14.3% of the variance in the motivation factors. The terms *job satisfaction *and *motivation *are often -but wrongly- used interchangeably in verbal (and often in written) communication; however there is a clear distinction between them. *Job satisfaction *is a person's emotional response to his or her job condition, whereas *motivation *is the driving force to pursue and satisfy needs. However, *job satisfaction *and *motivation *work together to increase *job performance *and healthcare organizations can do many things to increase *job satisfaction*, primarily by focusing on the motivating interests of existing and future staff [29].

To further interpret the motivational factors addressed in the present study, we attempt to link them to timeless motivational theories. Specifically, *remuneration *can be linked to the lower level of Maslow's needs pyramid (physiological and safety). The *co-workers *factor is equivalent to the third level of the pyramid described as social needs. The establishment of respect, trust and communication between co-workers is very important among these professional groups [30]. *Job attributes *has an apparent association with the fourth level (esteem) and the intrinsic factor *achievements *is linked to the highest level, self-actualization. According to Herzberg's two-factor theory, *remuneration *and *co-workers *are hygiene factors. While these do not motivate, they can satisfy if handled properly. On the other hand, factors *job attributes *and *achievement *are motivation factors because they create satisfaction by fulfilling an individual's higher needs. Once hygiene factors are met, the motivation factors will, according to Herzberg, promote job satisfaction and encourage better performance. However the link between the motivational factors and the above-mentioned theories presented in this paper is purely tentative and requires further substantiation in future studies.

The formulation of a structured personnel management strategy could have a positive impact on the quality of the services provided. Health care delivery is highly labor-intensive, and service quality, efficiency and equity are all directly related to providers' willingness to apply themselves to their tasks. Low motivation leads to the insufficient translation of knowledge, the underutilization of available resources and weak health system performance [31,32]. That the members of the three professional groups differed in their opinions as to what constituted the most important elements for their motivation implies that hospital managers should take these differences into account in their efforts for constructing effective human resource management strategies, as has been suggested elsewhere as well [33]. A limiting factor in this study might be the relatively small number of physicians participating. To increase this number, the survey could be carried out in the rest of the main hospitals on the island so that comparisons of the outcomes could be made and an integrated strategy formulated. Furthermore, the use of identical questions on different professions risks generating differing interpretations, but on the other hand it also allows direct comparison between professional groups, and therefore this methodology has been employed in other studies as well [18,34].

In conclusion, this study showed that motivation was influenced by both financial and non-financial incentives. The main motivating factors for the health workers in this public hospital sample were appreciation by managers and colleagues, a stable job/income and training. The main discouraging factors were related to low salaries and difficult working conditions. Activities associated with appreciation such as performance management are currently not optimally implemented, as health workers perceive supervision as control, selection for training as unclear and unequal and performance measurement as not useful. The kind of non-financial incentives identified should be taken into consideration when developing human resource management strategies. The knowledge of *motivation factors *and factors leading to increased *job satisfaction *allow the implementation of targeted strategies of continuous improvement [35].

## Competing interests

The authors declare that they have no competing interests.

## Authors' contributions

PL was responsible for conducting the literature review, acquiring and analyzing the data and drafting the manuscript. NK assisted in interpreting the results and finalizing the manuscript. DN was responsible for conception of the study and revising the manuscript for intellectual content. All authors have read and approved the final manuscript.
